# Screening of long-term complications and glycaemic control of patients with diabetes attending Rustenburg Provincial Hospital in North West Province, South Africa

**DOI:** 10.4102/phcfm.v5i1.375

**Published:** 2013-04-15

**Authors:** John M. Tumbo, Faustin N. Kadima

**Affiliations:** 1 Department of Family Medicine and Primary Health Care, University of Limpopo, Medunsa, South Africa

## Abstract

**Background:**

The prevalence of diabetes mellitus (DM) is increasing worldwide, with more than 90% being type 2. In South Africa, DM is common amongst all racial groups with the highest prevalence amongst the Indian population (15.8%), followed by the White (3.5%) and Black (4.8%) populations. Long-term cardiovascular, renal, neurovascular and retinal complications of type 2 DM are major causes of disability and mortality - hence the need for screening.

**Objective:**

To describe the screening practices of long-term complications amongst patients with type 2 diabetes attending Rustenburg Provincial Hospital in North West Province (South Africa).

**Method:**

A cross-sectional quantitative study using patients’ clinical records was performed. A random sample of 92 out of 1340 patients with type 2 diabetes attending the hospital in 2007 was selected. Demographic information on age, gender, body mass index, residence, level of education, duration of treatment and type of treatment was obtained. The recorded glycosylated haemoglobin (HbA_1_c), lipids and blood pressure levels were extracted, as well as the results of the dilated eye exam, foot examination, urine test for microalbumin, blood urea and creatinine. The data was analysed using the EPI Info version 6.05 software package.

**Results:**

The screening tests that were carried out consistently included: glycosylated haemoglobin (95.7%), blood pressure (100%), serum glucose (100%), serum cholesterol (79.3%) and serum creatinine (93.5%). Aspects poorly screened for were: dilated eye examination (19.5%), foot examination (20.6%), urine test for micro-albumin (1.1%), as well as HDL and LDL cholesterol (17.4%). Abnormal results were mainly detected in: HbA_1_c (69.3%), serum creatinine (30.2%), dilated eye examination (38.9%) and foot examination (52.6%). The HbA_1_c of 9.1% is far above the target of 6% and this predisposes patients to long-term complications.

**Conclusion:**

The screening of long-term complications of type 2 DM was poor in most patients and demonstrated a high prevalence of abnormal results. There is a need to improve screening practices.

## Abstrait

**Dépistage des complications à long terme et le contrôle glycémique des patients diabétiques participant Rustenburg hôpital provincial dans la province du Nord-Ouest, Afrique du Sud**

**Présentation:**

La prévalence de diabète non insulinodépendant (DNID) est en hausse dans le monde, plus de 90% des personnes affectées souffrant d'un diabète de type 2. En Afrique du Sud, le DNID est présent dans tous les groupes raciaux, la prévalence la plus élevée étant observée dans la population indienne (15,8%), suivie par les populations blanche (3,5%) et noire (4,8%). Les complications cardiovasculaires, rénales, neurovasculaires et rétiniennes à long terme du DNID de type 2 sont des causes majeures de handicap et de mortalité, d'où la nécessité de leur dépistage.

**Objectif de l’étude:**

Décrire les pratiques de dépistage des complications à long terme chez les patients souffrant d'un diabète de type 2 et consultant à l'hôpital provincial de Rustenburg, dans la province du Nord-ouest (Afrique du Sud).

**Méthode:**

Une étude transversale quantitative basée sur les dossiers médicaux des patients a été réalisée. Un échantillon aléatoire de 92 patients sur 1340 souffrant de diabète de type 2 et consultant à l'hôpital en 2007 a été sélectionné. Des informations démographiques telles que l’âge, le sexe, l'indice de masse corporelle, le lieu de résidence, le niveau d’éducation, la durée du traitement et le type de traitement ont été obtenues. L'hémoglobine glyquée (HbA_1_c) enregistrée, les niveaux de lipide et la pression artérielle ont été relevés, ainsi que les résultats de l'examen du fond de l'oeil, l'examen des pieds, les analyses d'urine destinées à contrôler les niveaux de microalbumine, d'urée sanguine et de créatinine. Les données ont été analysées à l'aide du logiciel EPI Info version 6.05.

**Résultats:**

Les tests de dépistage réalisés de manière systématique incluaient: l'hémoglobine glyquée (95,7%), la tension artérielle (100%), le glucose sérique (100%), le cholestérol sérique (79,3%) et la créatinine sérique (93,5%). Les points faisant l'objet d'un dépistage réduit étaient les suivants: l'examen du fond de l'oeil (19,5%), l'examen des pieds (20,6%), les analyses d'urine afin de détecter le niveau de microalbumine (1,1%), ainsi que le cholestérol LHD et le cholestérol LBD (17,4%). Des résultats anormaux ont essentiellement été détectés dans les analyses suivantes: HbA_1_c (69,3%), créatinine sérique (30,2%), examen du fond de l'oeil (38,9%) et examen des pieds (52,6%). Le taux de HbA_1_c de 9,1% est largement supérieur à l'objectif de 6%, ce qui prédispose les patients à des complications à long terme.

**Conclusion:**

Le dépistage des complications à long terme du DNID de type 2 était faible pour la plupart des patients et révélait une forte prévalence de résultats anormaux. Il est nécessaire d'améliorer les pratiques de dépistage.

## Introduction

Diabetes mellitus (DM) is a major public health problem in both developed and developing countries.^1^ In the USA, DM is a serious disease that affects over 16 million people (6% of the population) and over 150 000 people die from the disease and its complications annually.^2^ More than 2 million Canadians are estimated to have DM, where most of these cases are classified as type 2. The economic burden of diabetes and its complications in Canada is estimated to be between 4 and 5 billion US dollar a year.^3^

Similar trends are evident in developing countries south of the Sahara. Type 2 diabetes accounts for well over 90% of diabetes cases in Sub Saharan Africa, and population prevalence proportions ranged from 1% in rural Uganda to 12% in urban Kenya. The reported prevalence of type 1 diabetes was low and ranged from 4 per 100 000 in Mozambique to 12 per 100 000 in Zambia. The prevalence of gestational diabetes varied from 0% in Tanzania to 9% in Ethiopia. Proportions of patients with diabetic complications ranged from 7% – 63% for retinopathy, 27% – 66% for neuropathy and 10% – 83% for microalbuminuria.^4^ In South Africa, the prevalence of diabetes mellitus is about 15.8% in the Indian population, 3.5% in the White population and 4.8% – 6% in the Black population.^5^

DM is a group of metabolic diseases characterized by hyperglycemia resulting from absolute or relative deficiency of insulin secretion, insulin action or both, with a fasting plasma glucose ≥ 7 mmol/L or a plasma glucose ≥ 11 mmol/L two hours post 75 g oral glucose load on two or more occasions.^6, 7, 8^ The chronic hyperglycemia of diabetes mellitus is associated with long-term damage, dysfunction and failure of various organs, especially the eyes, kidneys, nerves, heart and blood vessels.^9, 10^ Type 2 DM, which accounts for 90% – 95% of all DM cases, may range from predominant insulin resistance with relative insulin deficiency to predominant insulin secretory defect with insulin resistance, unrestrained hepatic glucose production and other hormonal deficiencies.^11, 12, 13^

Chronic complications associated with long-term DM are devastating to the sufferers and lead to premature, increased mortality and morbidity. Adults with DM have an annual mortality rate double that of non-diabetic adults.^14^ According to Kalk, DM is responsible for almost 4000 deaths per year in South Africa - most of these deaths are middle-aged people and should be preventable.^14, 15^ A study conducted amongst black patients at a primary health care in the public sector of Cape Town showed a high prevalence of DM complications:

- Mean duration of diabetes: 8 (0–28)
- Retinopathy: 55.4%
- Cataracts: 7.9%
- Peripheral neuropathy: 27.6%
- Absent foot pulse: 8.2%
- Amputations: 1.4%
- Persistent proteinuria: 5.2%.

They concluded that complications from DM are neither identified nor managed well by family physicians.^16^

Studies have shown that most of the complications of DM can be slowed down or even prevented by better management on the part of the health care team, as well as the patient.^17, 18^ The complications are minimized through tight glycaemia control and regular screening in accordance with available guidelines. The Society of Endocrinology, Metabolism and Diabetes of South Africa (SEMDSA) developed screening guidelines that recommend tests to de conducted quarterly for glycated haemoglobin (HbA_1_c), monthly for blood pressure, blood glucose, body mass index and waist circumference measurement, as well as a yearly dilated eye examination, lipid profile, serum creatinine, electrocardiogram and urine test for microablumin.^19^

Bojanala is one of the four districts in North West Province, with about 40% of the 3.3 million being rural populations. DM is one of the four top chronic conditions in the Bojanala district, with an estimated prevalence of 19.5% amongst the adult population.^20^ Rustenburg Provincial Hospital is a 329 bed level 2 public referral hospital serving a population of about 349 000 and seeing about 650 patients with diabetes per month - with a high occurrence of complications. In the six months preceding this study, 5 cases of blindness and 4 cases of limp amputation due to DM were identified.^20^ This raised concerns about the screening practices implemented at this hospital and it was the motivation for the conduct of this study.

### Aim of the study

The aim of the study is to describe the screening practices for long-term complications of type 2 diabetes mellitus, such as retinopathy, nephropathy, foot problems and glycaemic control, amongst patients attending Rustenburg Provincial Hospital in North West Province, South Africa.

### Significance of the study

This study will provide baseline data on the screening practices of complications of diabetes that will be a valuable guide for interventions to improve the quality of care of patients and to prevent the complications.

## Ethical considerations

The Research, Ethics and Publications Committee (REPC) of the University of Limpopo (Medunsa Campus) granted ethical approval. The clearance certificate number is MP 107/2006. Permission for accessing patients’ records was obtained from the head of the institution. Confidentiality of patients’ information was maintained by excluding their names and identifiers.

## Methods

### Design

A descriptive cross-sectional study that reviewed clinical information contained in patients’ records was conducted in 2007. The study population comprised of all patients with diabetes attending the hospital outpatient clinic. From the sequentially numbered 1340 files, 92 patient files were systematically selected by taking every 15th file following random identification of the first one between 1 and 15.

### Procedure

Data collected from patient files included:

- The demographic characteristics: age, sex, residence (urban or rural), level of education and body mass index.
- Level of control recorded: glycaemic control, glycated haemoglobin (HbA_1_c), lipid (total cholesterol, LDL cholesterol and HDL cholesterol) and blood pressure goals.
- Documented tests and examinations for detection of long-term complications in the previous year: dilated eye exam, comprehensive foot exam and detection of nephropathy through urine dipstick, urine for micro albumin, urea and creatinine.

### Analysing

Data was captured and descriptive analysis performed by a statistician using EPI Info software, version 6.05.

## Results

Records of 92 patients with type 2 DM were reviewed and analysed. In this population, type 2 DM occurs mainly in females (69.6%), those aged over forty years (94.6%), those with a low education level (72.7%) and those living in rural areas (65.2%) (Table 1). The cut-off age of 40 for type 2 DM was chosen because it is the arbitrary age of distinction between type 1 and type 2 DM.^21, 22^

**TABLE 1:** Demographic and disease related characteristics.

Parameter	Categories	*N*	%
Age	< 40 years	5	5.4
	> 40 years	87	94.6
Sex	Male	28	30.4
	Female	64	69.6
Highest Education Level	None or Primary	67	72.8
	Secondary	25	27.2
	Tertiary	0	0
Residence	Urban	32	34.8
	Rural	60	65.2
Duration of diabetes Mellitus disease	< 10 years	68	73.9
	> 10 years	24	26.1
Obesity	Non-obese	53	57.3
	Obese	33	35.9
	Morbid obesity	6	6.5
Treatment modality	Oral anti-diabetic treatment only	36	41.3
	Insulin only	0	0
	Combination of insulin and oral treatment	46	50
			
**Total**		**8**	**8.6**

*N*, Given as number.

Most of the patients have been living with the disease for less than 10 years. The cut-off duration of 10 years was chosen following the observation that most long-term complications appear after 10 years of living with the disease.^23^ Obesity (either mild or morbid) was common with a prevalence of 42.4%. With regard to treatment, most patients (50%) were receiving insulin therapy instead of oral anti-diabetic agents (41.3%), where 8.7% received combination therapy.

Screening tests for complications were not preformed consistently. Some screening procedures, such as the dilated eye examination of the retina, was carried out on only 20.1% of the patients, whilst others, such as glycated haemoglobin and random glucose, were performed as often as 100% of the time (Table 2). The highest proportion of abnormal results was found in glycated haemoglobin (70.4%) and high-density lipoproteins (72%).

**TABLE 2:** Screening tests for complications.

Screening test	Participants with test performed		Abnormal results	
		
	*N*= 92	%	*N*	%
Glycated haemoglobin (HbA_1_c)	88	95.6	62	70.4
Dilated eye exam	19	20.1	7	38.8
Comprehensive foot exam	20	21.6	9	47.4
Blood pressure	92	100	47	50.1
Random glucose	92	100	27	29.3
Total cholesterol	74	80.4	19	25.6
High density lipoprotein	25	27.1	18	72
Low density lipoprotein	25	27.1	5	20
Urine for micro albumin	1	1.1	0	0
Serum creatinine	86	93.4	26	30.2

*N*, Given as number.

## Discussion

The main finding of the study was the low frequency of screening of long-term complications of DM in the majority of patients, particularly for retinopathy (19.5%), nephropathy (1.11%) and diabetic foot problems (20.6%). These are tests that require clinical or technical skills to perform. This study also showed that tests requiring less technical skills, such as glycated haemoglobin, blood pressure, random blood glucose, total cholesterol and serum creatinine, were frequently performed and recorded.

Abnormal results were found in 42.1% (220 out of 522) of all screening tests performed for long-term complications.

Diabetic retinopathy is one of the most prevalent, but preventable causes of blindness. Several randomized controlled trials, such as the DCCT trial, showed that intensive blood glucose control reduced the risk of the progression of diabetic retinopathy by 54%, reduced the development of severe non-proliferative diabetic retinopathy or proliferative diabetic retinopathy by 47%, reduced the need for laser surgery by 56% and reduced the risk of diabetic macular oedema by 23%.19 Because of the low frequency of screening of retinopathy at RPH (19 out of 92), it is difficult to draw conclusive inference from this aspect of the study. According to the SEMDSA guidelines, all patients were supposed to have had one retina examination in the previous year.

In the current study, diabetic nephropathy was screened by the means of serum creatinine and microalbuminuria. Using urine for micro albumin level carried out in the past year, the study showed very poor results, since only 1 out of the 92 patients (1.1%) was screened. Serum creatinine levels were tested in 93.5% (86 out of 92) of the patients and 30.2% had results indicative of nephropathy. The presence of microalbuminuria predicts the worsening of renal disease to overt diabetic nephropathy and an elevated risk of cardiovascular disease.^24^ A urine test for micro albumin is an essential test that detects early signs of nephropathy, but it was infrequently preformed at this hospital that offered a wide range of primary health care services. A study carried out in a setting in Hungary providing similar services, demonstrated a higher frequency of screening for microalbuminuria. Amongst patients with diabetes in the primary health care system in Hungary, Forkas showed that microalbuminuria was detected in 24.8% of type 1 DM patients and 25% of type 2 DM patients.^25^ The study concluded that one third (33.8%) of patients with diabetes in a primary care setting exhibited signs or were at risk of renal involvement of diabetes.

Foot complications are common in patients with diabetes and are considered one of the most devastating diabetic complications. The current study showed foot complications were screened in only 20.7% of participants. Amongst those screened, slightly less than half (47.4%) had abnormal results. This was much higher than that found at a primary care clinic in Germany, where the prevalence of foot abnormalities was estimated to be 2.9% in type 2 DM patients with almost 50% of patients with diabetic foot problems having major and minor amputations.^26^ In Moscow, this prevalence was shown to be 11.22% in type 1 DM and 5.58% in type 2 DM^27^.

Complications from diabetes can often be prevented or delayed with good primary care, screening for complications and compliance with the advice from health care providers.17 In the current study, the target of HbA_1_c was based on the recommendations of SEMDSA and was less than 8%.^18^ This study showed that 95.7% of patients had their HbA_1_c tested and a high percentage (69.3%) had abnormal results (did not achieve glycaemic control). The average HbA_1_c of 9.1% of the patients in the study was above the recommendation of SEMDSA (7% – 8%) and this could predispose patients to longterm complications.

### Limitations of the study

The cross-sectional study design allowed the researchers to only obtain a snap shot of the situation regarding screening patterns of long-term complications of type 2 DM at RPH at that particular time. A prospective study would have been beneficial in capturing information that will help to estimate the prevalence of long-term complications of type 2 DM patients.

The use of patients’ records as a source of data could be a limitation because of the poor documentation and the inability to differentiate the stages of complications, such as retinopathy. The results analysed only documented normal and abnormal results without specification of anatomical lesion and severity.

This study focused on the screening practices at a referral hospital. It could not provide information on screening practices at primary health care facilities where the bulk of patients with diabetes and other chronic illnesses are managed.

## Conclusion and recommendations

The screening of long-term complications of type 2 DM, such as retinopathy, nephropathy and foot problems, was poor in the majority of patients. The screening tests performed showed a high percentage of abnormal results. The poor screening practices could explain the increase in the late identification of long-term complications. To address this problem in the local context, it is recommended that screening protocols be implemented at all treatment points, that clinicians are trained on the protocols and skills for the screening, and lastly that a quality improvement project be immediately conducted using this data as the baseline.

## References

[1] Rheeder P Type 2 diabetes: The emerging epidemic. S Afr Fam Pract. 2006;48(10):20.

[2] American Diabetes Association Screening for type 2 diabetes. Diabetes care. 2000;23:20–23.

[3] Harris SB, Petrella RJ, Leadbetter W Lifestyle interventions for type 2 Diabetes Mellitus. Relevance for clinical practice. Can Fam Physician. 2003;49:1618–1625. PMid:14708927, PMCid:2214163 14708927 PMC2214163

[4] Hall V, Thomsen RW, Henriksen O, Lohse N Diabetes in Sub Saharan Africa 1999–2011: epidemiology and public health implications. A systematic review. BMC Public Health. 2011 14;11:564. 10.1186/1471-2458-11-564 PMC3156766 21756350

[5] Seedat YK Implementing the South African hypertensive guidelines in diabetes. SAJDVD. 2006;3(2):53.

[6] Haslett C, Bonn AN, Chilvers E Diabetes Mellitus Davidson's Principles of Medicine. 19th ed Edinburg: Churchill, 2002; p. 642–682.

[7] Huddle KRL 2005 Practical diabetes management. WITS Diabetes group.

[8] Herman WH Glycaemic control in diabetes. Clinical evidence. BMJ. 1999;319:104–106. http://dx.doi.org/10.1136/bmj.319.7202.104, PMid:10398638, PMCid:1116194 10398638 10.1136/bmj.319.7202.104 PMC1116194

[9] American Diabetes Association Screening for type 2 diabetes. Diabetes care. 2000;23:20–23.

[10] Mayfield J Diagnosis and classification of diabetes mellitus: New criteria Am Acad of Fam Physician; 1998. 9803200

[11] Rhedeer P Outpatient diabetes management. University of Pretoria booklet; 2004.

[12] American Association of Clinical Endocrinologists Medical Guidelines for Clinical Practice for the Management of Diabetes Mellitus; 2007. 10.4158/EP.13.S1.1 17613449

[13] Mbokazi AJ &Ndwamato N Diabetes mellitus: Chronic complications. CME. 2006;24(10);558–566.

[14] Kalk WJ, Pick WM, Sayed AR Diabetic Mortality in South Africa. SAMJ. 1998;88: 1259–1262.

[15] Levitt NS, Bradshaw D, Zwarenstein MF, Maphumolo S Audit of public sector primary diabetes care in Cape Town, South Africa: high prevalence of complications, uncontrolled hyperglycemia, and hypertension. Diabetic Med. 1997;14:1073–1077. http://dx.doi.org/10.1002/(SICI)1096-9136(199712)14:12@1073:AID-DIA498#3.0.CO;2-9 9455936 10.1002/(SICI)1096-9136(199712)14:12<1073::AID-DIA498>3.0.CO;2-9

[16] Centre for Disease Control and Prevention National diabetes fact sheet, national estimates and general information on diabetes in the United States. Revised edition Atlanta, GA; US Department of Health and Human Resources; 1998.

[17] Diabetes care Summary of revisions for the 2005 clinical practice recommendations. ADA. 2005;28:S3.

[18] Revised Society for Endocrinology, Metabolism, and Diabetes of South Africa (SEMDSA) Guidelines for diagnosis and management of type 2 diabetes mellitus for primary health care in 2002.

[19] DCCT Research Group (1995). The relationship of glycaemic exposure (HbA1c) to the risk of development and progression of retinopathy in the diabetic control and complications trial. Diabetes http://dx.doi.org/10.2337/diabetes.44.8.968

[20] Chronic Disease Register Bojanala District Health Information System; 2007.

[21] Ogunbanjo GA 2006. Type 2 Diabetes: An evidence-based approach to its management by family practitioners. CME 2006;24(10):568–572.

[22] American Diabetes Association Diagnosis and classification of diabetes mellitus. Diabetes care. 2008;31:S55–S60. http://dx.doi.org/10.2337/dc08-S055, PMid:18165338 18165338 10.2337/dc08-S055

[23] Maberley D, Walker H, Koushik A, Cruess A Screening for diabetic retinopathy in James Bay, Ontario: a cost-effectiveness analysis. CMAJ. 2003;168(1):160–164. 12538543 PMC140424

[24] Tobe SW, McFarlane PA, Naimark DM Microalbuminuria in diabetes mellitus. CMAJ. 2002;167(5). PMid:12240818, PMCid:121969 PMC121969 12240818

[25] Forkas K Screening of microalbuminuria in diabetic patients in the primary health care system. Orv Hetil. 1997;138(8):459–465. 9139249

[26] Sämann A, Tajiyeva O, Muller N Prevalence of the diabetic foot syndrome at the primary care level in Germany: a cross sectional study. Diabetic med. 2001;25(5): 557–563. http://dx.doi.org/10.1111/j.1464-5491.2008.02435.x, PMid:18346154 18346154 10.1111/j.1464-5491.2008.02435.x

[27] Bakharev IV, Misnikova IV, Dreval AV Prevalence of diabetic foot syndrome among diabetic patients. European Congress of Endocrinology 2006. Endocrine abstracts 2006;11:339.

